# Keys to the avian malaria parasites

**DOI:** 10.1186/s12936-018-2359-5

**Published:** 2018-05-29

**Authors:** Gediminas Valkiūnas, Tatjana A. Iezhova

**Affiliations:** Nature Research Centre, Akademijos 2, 08412 Vilnius 2100, Lithuania

**Keywords:** Avian malaria, Key to species, *Plasmodium*, Species inquirenda, Synonym, Avian *Plasmodium* taxonomy

## Abstract

**Background:**

Malaria parasites (genus *Plasmodium*) are widespread in birds. These pathogens cause pathology of blood and various organs, often resulting in severe avian malaria. Numerous recent studies have reported DNA sequences of avian malaria parasites, indicating rich genetic diversity and the possible existence of many undescribed species. However, the majority of reported *Plasmodium* lineages remain unidentified to species level, and molecular characterization is unavailable for the majority of described *Plasmodium* parasites. During the past 15 years, numerous new *Plasmodium* species have been described. However, keys for their identification are unavailable or incomplete. Identification of avian malaria parasites remains a difficult task even for experts, and this precludes development of avian malariology, particularly in wildlife. Here, keys for avian malaria parasites have been developed as a baseline for assisting academic and veterinary medicine researchers in identification of these pathogens. The main obstacles and future research priorities have been defined in the taxonomy of avian *Plasmodium* species.

**Methods:**

The data were considered from published articles and type and voucher material, which was accessed in museums in Europe, the USA and Australia. Blood films containing various blood stages of the majority of described species were examined and used for the development of dichotomous keys for avian *Plasmodium* species.

**Results:**

In all, 164 published articles were included in this review. Blood stages of avian *Plasmodium* parasites belonging to subgenera *Haemamoeba*, *Giovannolaia*, *Novyella*, *Bennettinia* and *Huffia* were analysed and compared. Illustrated keys for identification of subgenera and species of these parasites were developed. Lists of invalid and synonymous *Plasmodium* parasite names as well as names of doubtful identity were composed.

**Conclusion:**

This study shows that 55 described species of avian *Plasmodium* can be readily identified using morphological features of their blood stages. These were incorporated in the keys. Numerous synonymous names of *Plasmodium* species and also the names belonging to the category *species inquirenda* exist, and they can be used as reserves for future taxonomy studies. Molecular markers are unavailable for 58% of described *Plasmodium* parasites, raising a task for the current avian malaria researchers to fill up this gap.

## Background

Malaria parasites of the genus *Plasmodium* (Haemosporida, Plasmodiidae) inhabit all major groups of terrestrial vertebrates. Avian malaria parasites is a peculiar group among them, particularly due to the ability of numerous species to develop and complete life cycles in numerous bird species belonging to different families and even orders [1–7]. The same is true for invertebrate hosts (vectors) of these parasites [8, 9]. Many species of avian *Plasmodium* use Culicidae mosquitoes belonging to different genera (*Culex, Coquillettidia, Aedes, Mansonia, Culisetta, Anopheles, Psorophora*) for completing sporogony and transmission [1, 8–11]. This is not the case in mammalian malaria parasites whose are transmitted mostly by *Anopheles* species [1, 12–14]. Furthermore, sporogony of many avian *Plasmodium* parasites is completed relatively fast in susceptible vectors at relatively low temperatures [1, 8, 15, 16]. These features likely contributed to the global distribution of some avian malaria infections, which are actively transmitted in countries with warm and cold climates, including regions close to the Polar Circles [6, 17–19].

Life cycles of avian malaria parasites are similar in their basic features to those of human and other mammal *Plasmodium* species [1, 2, 8, 13, 14, 20]. Malaria parasites are obligate heteroxenous protists, with merogony in cells of fixed tissues and also blood cells. Gametogony occurs in red blood cells, and sexual process and sporogony are completed in Culicidae mosquitoes. However, the life cycles of avian *Plasmodium* species differ from those of the parasites of mammals, particularly due to their relatively low host specificity and marked variation in patterns of development in avian hosts and vectors. For example, *Plasmodium (Haemamoeba) relictum* infects and completes its life cycle in birds belonging to over 300 species and 11 orders, and *Plasmodium (Huffia) elongatum*, *Plasmodium (Novyella) vaughani* and many other species also have a broad range of avian hosts [6, 8, 21–23]. Erythrocytic merozoites of many avian malaria parasites can induce secondary tissue merogony in birds [24, 25]. The exo-erythrocytic merogony occurs in cells of the reticuloendothelial and haemopoietic systems, but has not been reported in hepatocytes [2, 4, 8, 23, 26]. Pedunculated oocysts were discovered in *Plasmodium (Bennettinia) juxtanucleare*; these oocysts possess leg-like outgrowths which attach the oocysts to the mosquito midgut wall [27]. These and some other features are not characteristics of malaria parasites of mammals, and this is reflected in genetic differences between these groups of parasites and their different position in molecular phylogenies [28–33].

Malaria, the disease caused by parasites of the genus *Plasmodium*, has traditionally been viewed as a disease of the blood and blood forming tissues of vertebrate hosts, with exo-erythrocytic stages of development causing little or no pathology [1, 13, 14, 34]. While available evidence still supports this view for the primate and rodent malarial parasites, there is increasing evidence that the pathogenicity of tissue stages of avian species of *Plasmodium* has been significantly underestimated [25]. Even more, avian malaria is often a more severe disease than human malaria. There is recent experimental evidence of unexpected pathology associated with obstructive development of secondary exo-erythrocytic stages of *Plasmodium* in brain capillaries that can lead to ischaemia and rapid death in birds that have very low intensity parasitaemias during chronic stage of infection [24, 25, 35]. Importantly, the severity of disease caused by a given lineage of *Plasmodium* often varies markedly in different species of avian hosts, from absence of any clinical symptoms to high mortality [4, 17, 19, 36–41].

Because of broad vertebrate host specificity, the same *Plasmodium* species can infect distantly related birds. In other words, vertebrate host identity cannot be used as a taxonomic feature during identification of avian malaria parasites [1, 12, 42]. This raises questions about parasite species identification if the same pathogen is found in unusual avian hosts. Molecular characterization is helpful in diagnosis of malaria infections, and has been developed for detection of some avian *Plasmodium* species [21, 40]. Molecular markers are essential in diagnosis and identification of exo-erythrocytic and vector stages, which cannot be identified using morphological features [11, 43, 44]. However, molecular diagnostics using general primers (the main diagnostic tool currently used in wildlife malariology) is often insensitive in distinguishing of avian *Plasmodium* spp. co-infections, which are common and even predominate in many bird populations [45–48]. Specific molecular markers for the majority of avian *Plasmodium* species have not been developed, and currently are difficult to develop due to significant genetic diversity of malaria parasites, which remain undescribed in wildlife. Morphological identification using microscopic examination of blood films remains important in malaria diagnostics in the wild, and is particularly valuable if it is applied in parallel with polymerase chain reaction (PCR)-based diagnostic tools [5, 30, 49, 50].

During the past 15 years, numerous avian *Plasmodium* parasites were named and described using morphological features of their blood stages [49, 51–59]. However, molecular markers for parasite detection were developed in a handful of these descriptions. The keys that are available for identification of avian *Plasmodium* species [8], should be reworked in the light of the newly available information.

The main aim of this review is to develop easy-to-use keys for identification of avian malaria parasites using morphological features of their blood stages as a baseline for assisting academic and veterinary medicine researchers in identification of these pathogens. Lists of synonymous names of *Plasmodium* species as well as invalid species names were updated and compiled. The *Plasmodium* parasite names of unknown taxonomic position (*incertae sedis*) and the species of doubtful identity requiring further investigation (*species inquirenda*) were specified as well. The information about useful molecular markers, which can be used for described *Plasmodium* species detection and comparison was also summarized. This review might be helpful for wildlife malaria and veterinary medicine researchers aiming identification of avian malaria infections.

## Methods

Full-length papers with descriptions of new *Plasmodium* species published in peer-reviewed journals were considered. In all, 164 articles were reviewed, and 152 papers containing most representative information about taxonomy of these parasites were incorporated in the References.

Type and voucher preparation as well as images of blood stages of avian *Plasmodium* parasites were obtained from the collections of Natural History Museum (London, UK), International Reference Centre for Avian Haematozoa (Queensland Museum, Quensland, Australia), the US National Parasite Collection (National Museum of Natural History, Washington DC, USA), Muséum National d’Histoire Naturelle (Paris, France), Grupo de Estudio Relación Parásito Hospedero, Universidad Nacional de Colombia (Bogotá, Colombia) and Nature Research Centre (Vilnius, Lithuania). All accessed preparations were studied. An Olympus BX61 light microscope (Olympus, Tokyo, Japan) equipped with an Olympus DP70 digital camera and imaging software AnalySIS FIVE (Olympus Soft Imaging Solution GmbH, Münster, Germany) was used to examine preparations and prepare illustrations.

A method of dichotomous key was applied for identification of *Plasmodium* species. This tool consists of steps divided it two alternative parts, which allow to determine the identity of a specimen due to a series of choices that lead the user to the correct name of a given specimen. The most difficult choices, which do not exclude ambiguity, were accompanied with references to the corresponding pictures, which illustrate meaning of the text information. This simplifies the comparison of diagnostic features used in the keys. All parasite names in the keys are accompanied with references to the original parasite descriptions and (or) reviews containing description and (or) illustrations of corresponding species.

## Results

**Fig. 1:**
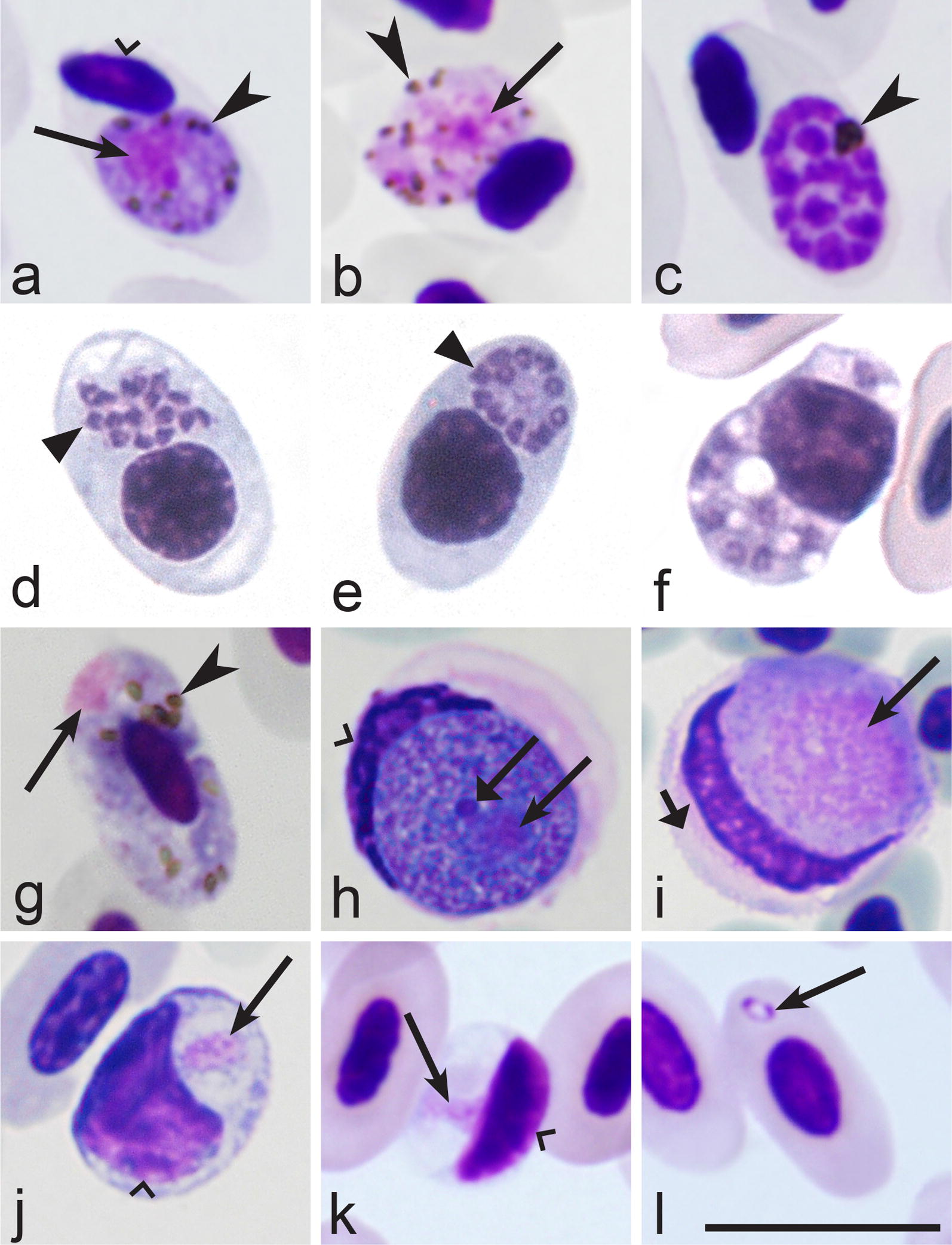
Main morphological features of blood stages, which are used for identification of families of haemosporidian (Haemosporida) parasites (**a**–**i**). Mature gametocytes (**a**, **b**, **g**–**i**) and meronts (**c**–**f**) of *Plasmodium* (**a**–**c**), *Garnia* (**d**, **e**), *Fallisia* (**f**), *Haemoproteus* (**g**) and *Leucocytozoon* (**h**, **i**) parasites belonging to the families Plasmodiidae (**a**–**c**), Garniidae (**d**–**f**), Haemoproteidae (**g**) and Leucocytozoidae (**h**, **i**). Note presence of malarial pigment in species of Plasmodiidae (**a**–**c**) and Haemoproteidae (**g**) and its absence in species of Garniidae (**d**–**f**) and Leucocytozoidae (**h**, **i**). Macrogametocytes (**a**, **g**, **h**) and microgametocytes (**b**, **i**) are readily distinguishable due to presence of sexually dimorphic features. Common avian intracellular non-haemosporidian parasites (**j**–**l**) are shown for comparison with haemosporidians. These are *Isospora* (synonym *Atoxoplasma*) (**j**), *Hepatozoon* (**k**) and *Babesia* (**l**). Long simple arrows—nuclei of parasites. Simple arrowhead—pigment granules. Triangle arrowheads—developing merozoites. Long simple wide arrow—nucleolus. Simple wide arrowheads—host cell nuclei. Short simple wide arrow—cytoplasm of host cell. Scale bar = 10 µm. Explanations are given in the text

Birds are often infected with different blood parasites belonging to same and different genera in the wild, and various combinations of different parasite co-infections often occur in same individual hosts. Haemosporidians (order Haemosporida) develop intracellularly, and they should be distinguished from other eukaryotic intracellular infections before identification of the parasite species identity. Haemosporidians can be readily distinguished from all other intracellular protists (species of *Babesia, Isospora, Lankesterella, Haemogrerina, Hepatozoon, Toxoplasma*) due to one particularly readily distinguishable feature. Mainly, gametocytes of all haemosporidians are characterized by sexually dimorphic features, which are readily distinguishable under the light microscope. Haemosporidian macrogametocytes possess compact nuclei and bluish-stained cytoplasm, and the microgametocyte nuclei are diffuse and the cytoplasm stains paler than in macrogametocytes (compare Fig. 1a, h with b, i). Some variation occurs in the size of nuclei and in the staining of the cytoplasm in different haemosporidian species. While, this also depends on staining protocols, macro- and microgametocytes can be readily distinguished in each haemosporidian species. This is not the case in other intracellular protists, whose gamonts and other intracellular blood stages do not show sexually dimorphic features and all look similar under the light microscope (Fig. 1j–l).

Based on current taxonomy, four families of haemosporidians can be recognized. These are Plasmodiidae, Haemoproteidae, Leucocytozoidae and Garniidae [1, 4, 8, 30, 60, 61]. Malaria parasites are classified in the family Plasmodiidae, which contains one genus *Plasmodium*. When haemosporidians are found in blood films, *Plasmodium* parasites should be distinguished from species of related haemosporidians belonging to the families Garniidae, Haemoproteidae and Leucocytozoidae. The main distinctive features of parasites belonging to these families are summarized in Table 1.

**Table 1:** Key to families of haemosporidian parasites

Step	Features and family	
1 (4)	Merogony takes place in blood cells (Fig. 1c–f)	
2 (3)	Malarial pigment (hemozoin) is present in blood stages (Fig. 1a–c)	
	…………………………………………… *Plasmodiidae*^a^	
3 (2)	Malarial pigment (hemozoin) is absent from blood stages (Fig. 1d–f)	
	…………………………………………… *Garniidae*^b^	
4 (1)	Merogony (Fig. 1c–f) does not take place in blood cells. Only gametocytes (Fig. 1g–i) present in blood cells	
5 (6)	Malarial pigment (hemozoin) is present in blood stages (Fig. 1a, b, g)	
	…………………………………………… *Haemoproteidae*^c^	
6 (5)	Malarial pigment (hemozoin) is absent from blood stages (Fig. 1h, i)	
	…………………………………………… *Leucocytozoidae*^d^	

Main taxonomic features of families of the haemosporidian parasites [8]

^a^Merogony takes place in cells of fixed tissues and blood cells of vertebrate hosts. Malarial pigment (hemozoin) is present in erythrocytic meronts and gametocytes. Sexual process and sporogony of bird parasites take place in mosquitoes (Diptera: Culicidae)

^b^Merogony takes place in cells of fixed tissues and blood cells of vertebrate hosts. Malarial pigment (hemozoin) is absent at all stages. Vectors are still unknown

^c^Merogony takes place in cells of fixed tissues of vertebrate hosts. No merogony occurs in blood cells. Malarial pigment (hemozoin) is present in gametocytes. Sexual process and sporogony of bird parasites take place in louse flies (Hippoboscidae) and biting midges (Ceratopogonidae)

^d^Merogony takes place in cells of fixed tissues of vertebrate hosts. No merogony occurs in blood cells. Malarial pigment (hemozoin) is absent at all stages. Sexual process and sporogony take place in black flies (Simuliidae) and biting midges (Ceratopogonidae)

Blood stages of species of *Plasmodium* are particularly similar to those of relatively rare haemosporidian parasites of the genera *Fallisia* and *Garnia* of the family Garniidae [8, 60–62]. Parasites of these three genera produce gametocytes and meronts (=schizonts) in blood cells (Fig. 1a–f). However, species of *Plasmodium* do not digest haemoglobin completely and accumulate residual pigment granules (hemozoin), which are refractory and readily visible in blood stages under light microscope (Fig. 1a–c). This is not true of species belonging to the genera *Fallisia* and *Garnia* or other garniids, which digest haemoglobin completely when they inhabit red blood cells and do not possess pigment granules in their blood stages (Fig. 1d–f).

When malaria parasites of the *Plasmodium* genus are reported in blood films, the next step is to distinguish subgenera of this genus. The main characteristics of different subgenera are summarized in Table 2.

**Table 2:** Key to subgenera of *Plasmodium* parasites of birds

Step	Features and subgenus	
1 (2)	Exo-erythrocytic merogony takes place in cells of the haemopoietic system. Erythrocytic meronts develop in various immature red blood cells (Fig. 2i, k–p)	
	…………………………………………… *Huffia*^a^	
2 (1)	Exo-erythrocytic merogony does not takes place in cells of the haemopoietic system. Erythrocytic meronts do not develop in early immature red blood cells (Fig. 2i, k–p); mature and nearly mature erythrocytes are the main host cells (Figs. 2a–h, j; 3a–y)	
3 (6)	Roundish fully grown gametocytes (Fig. 4t–w) are present	
4 (5)	Size of fully grown gametocytes (Fig. 4u–w) and erythrocytic meronts (Fig. 1c) markedly exceed that of the nuclei of infected erythrocytes	
	…………………………………………… *Haemamoeba*^b^	
5 (4)	Size of fully grown gametocytes (Figs. 4a, 5m) and erythrocytic meronts (Fig. 3g, n) does not exceed that of the nuclei of infected erythrocytes	
	…………………………………………… *Bennettinia*^c^	
6 (3)	Roundish fully grown gametocytes (Fig. 4t–w) are absent. Elongate gametocytes (Fig. 4c–s) predominate	
7 (8)	Erythrocytic trophozoites (Fig. 3d) and growing meronts (Fig. 3x) contain plentiful cytoplasm. Size of fully grown erythrocytic meronts, which size markedly exceed that of the nuclei of infected erythrocytes (Figs. 2d, e, 3w, y), are present	
	…………………………………………… *Giovannolaia*^d^	
8 (7)	Erythrocytic trophozoites (Fig. 3a, b) and growing meronts (Fig. 3e–o) contain scanty cytoplasm. Size of fully grown erythrocytic meronts does not exceed or only slightly exceeds that of the nuclei of infected erythrocytes (Fig. 3p–r)	
	…………………………………………… *Novyella*^e^	

Main taxonomic characters of subgenera of avian malaria parasites [8]

**Fig. 2:**
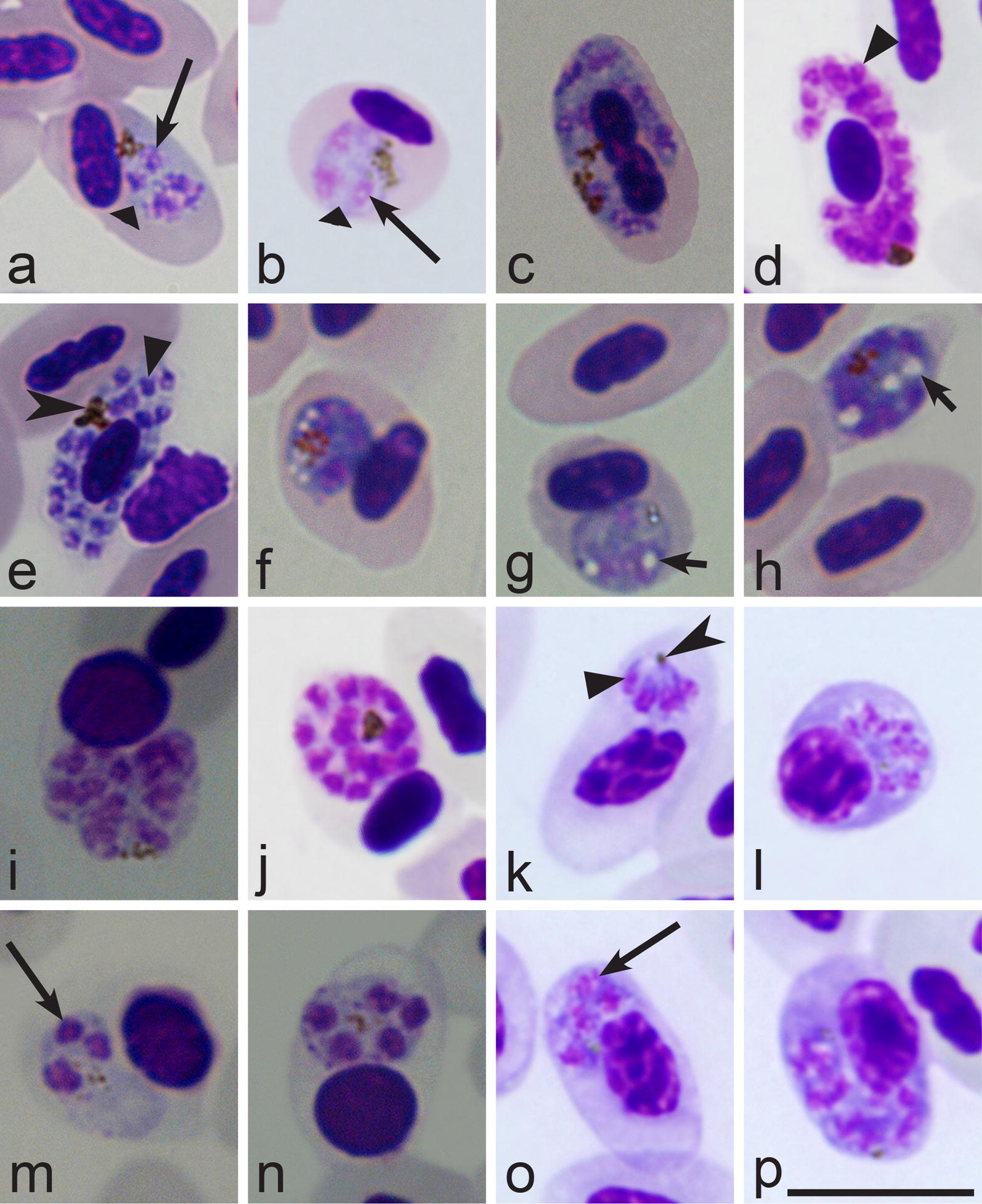
Morphological features of erythrocytic meronts and their host cells of avian *Plasmodium* parasites, which are used for *Haemamoeba*, *Giovannolaia* and *Huffia* species identification. Growing (**a**–**c**, **f**–**h**, **l**–**p**) and mature (**d**, **e**, **i**–**k**) meronts at different stages of their development. Note presence of the plentiful cytoplasm and large nuclei in early growing meronts (**a**, **b**, **f**–**h**, **m**–**p**), marked vacuolization of the cytoplasm (**f**–**h**), elongate shape of mature merozoites (**k**), presence of meronts in erythroblasts (**i**, **l**–**n**) and other immature red blood cells (**k**, **o**, **p**), and distinct smooth outline in growing erythrocytic meronts (**m**, **n**). Short simple arrows—vacuoles. Wide triangle arrowheads—the cytoplasm. Other symbols are as in Fig. 1. Explanations are given in the text

^a^Exo-erythrocytic merogony takes place in cells of the haemopoietic system. Erythrocytic trophozoites and growing meronts (Fig. 2l–p) contain plentiful cytoplasm. Erythrocytic meronts develop in various immature red blood cells (*Plasmodium huffi* probably is an exception, but this needs confirmation). Fully grown erythrocytic meronts and gametocytes are variable both in form and size; elongate, roundish and irregularly shaped parasites might occur. Pedunculated oocysts are absent

**Fig. 3:**
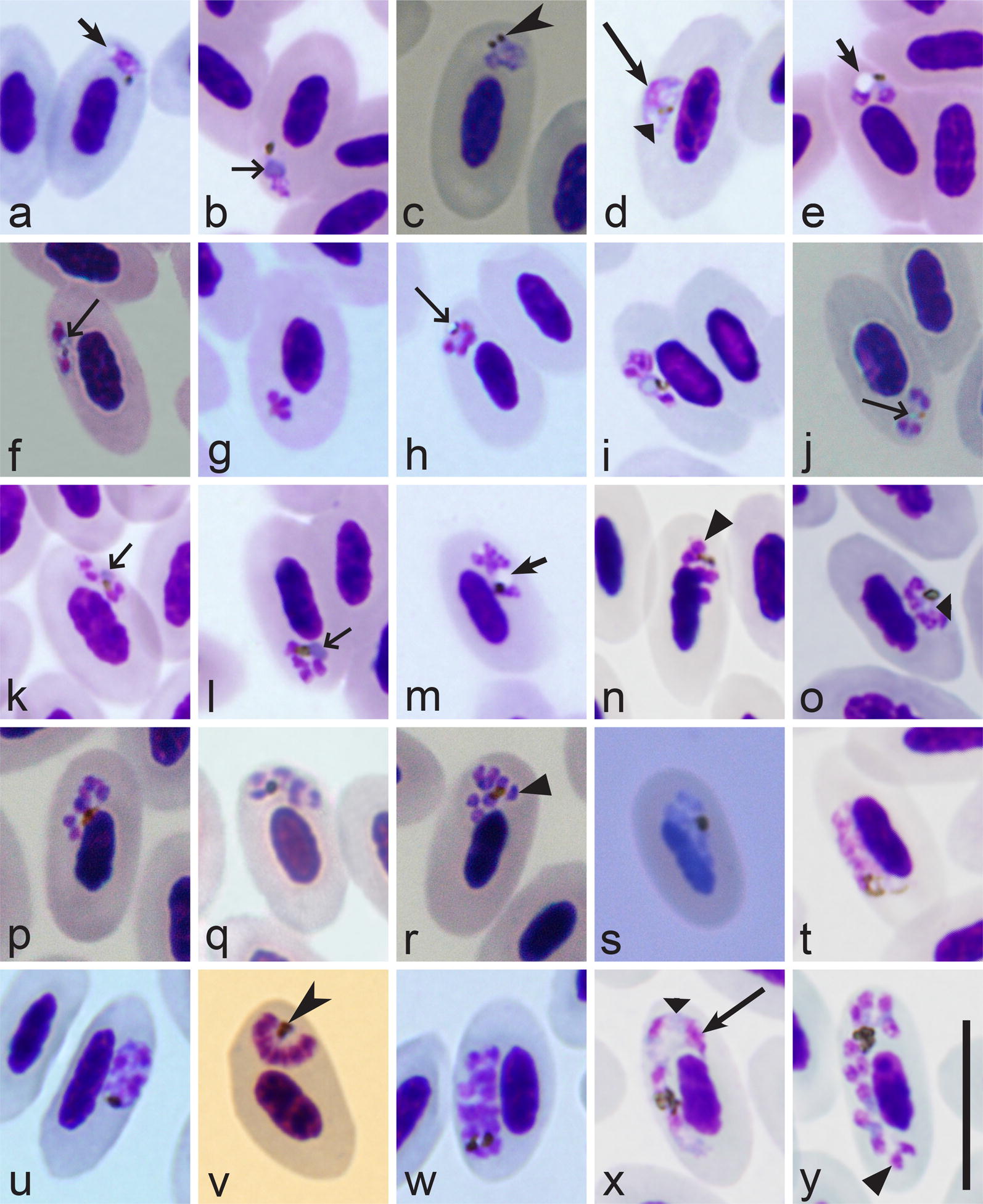
Morphological features of erythrocytic meronts and their host cells of avian *Plasmodium* parasites, which are used for *Novyella* and *Giovannolaia* species identification. Trophozoites (**a**–**d**) and erythrocytic meronts (**e**–**y**) on different stages of maturation. Note presence of large vacuoles (**a**, **e**, **m**), refractive small globules (**f**, **h**–**j**), bluish non-refractive globules (**b**, **k**, **l**), fan-like mature meronts (**o**, **v**), strictly nucleopilic position (**n**, **t**), the scanty (nearly invisible) cytoplasm (**a**, **b**, **e**–**l**) and the prominent (readily visible) cytoplasm (**d**, **x**) in parasites on different stages of their development. Triangle wide long arrows—refractive globules. Triangle wide short arrows—bluish (non-refractive) globules. Other symbols are as in Fig. 1. Explanations are given in the text

**Fig. 4:**
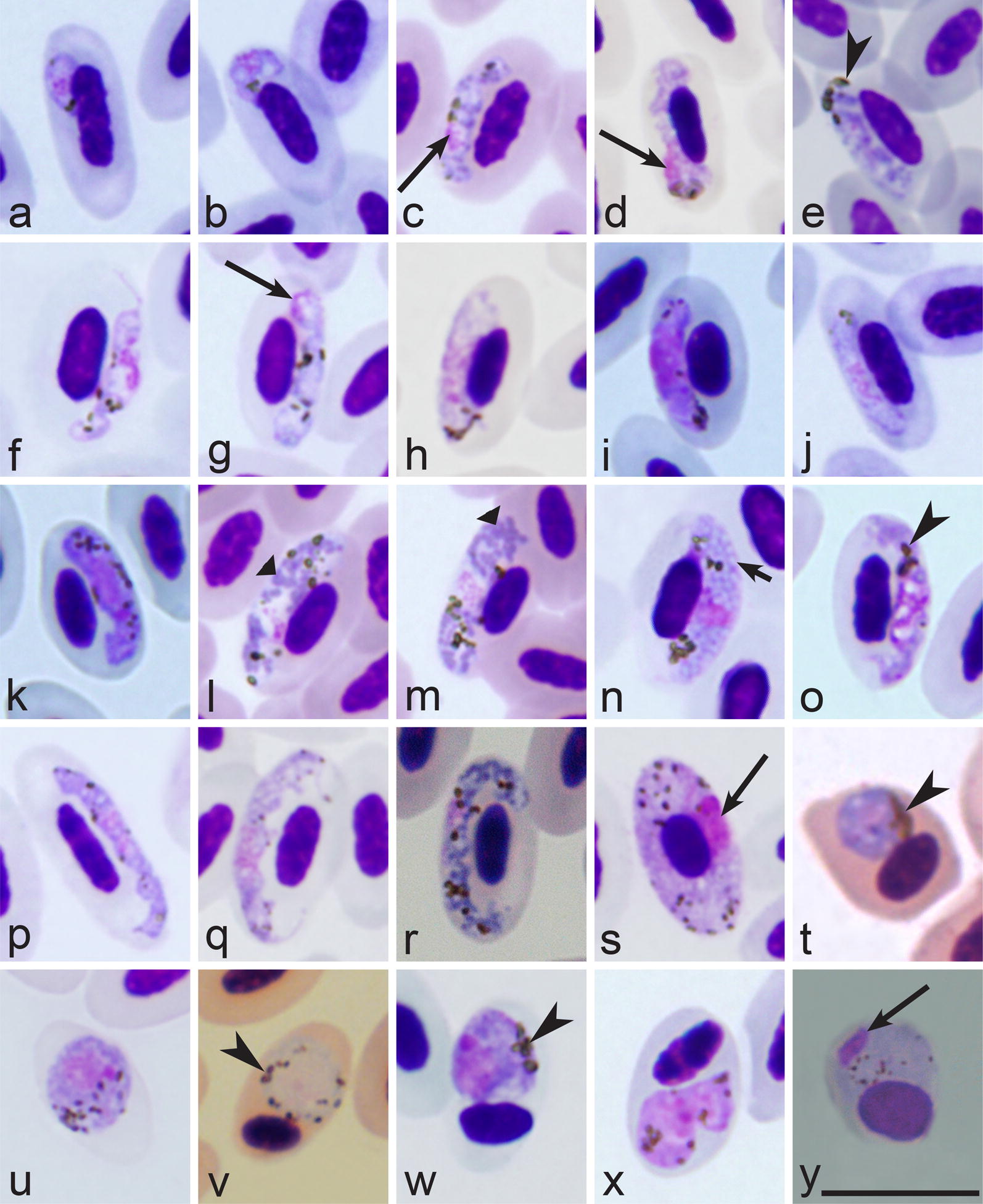
Morphological features of gametocytes and their host cells of avian *Plasmodium* parasites, which are used for species identification. Macrogametocytes (**a**–**g**, **k**–**u**, **w**–**y**) and microgametocytes (**h**–**j**, **v**). Note long outgrowth (**f**), terminal position of pigment granules (**e**) and nucleus (**g**), granular (**l**, **m**) and vacuolated (**n**) appearance of the cytoplasm, slender (**p**–**r**) and circumnuclear (**s**) shapes of gametocytes, clumps of pigment granules located near the parasite margin (**t**, **w**), distinct smooth outline of nucleus (**y**). Symbols as in Figs. 1, 2, 3. Explanations are given in the text

^b^Exo-erythrocytic merogony takes place in cells of the reticuloendothelial system. Erythrocytic trophozoites (Fig. 3d) and growing meronts (Fig. 2a, b) contain plentiful cytoplasm. The size of fully grown erythrocytic meronts exceeds that of the nuclei of infected erythrocytes (Fig. 1c). Fully grown gametocytes are roundish, oval or of irregular form, and their size exceeds that of the nuclei of infected erythrocytes (Fig. 4t–x). Pedunculated oocysts are absent

**Fig. 5:**
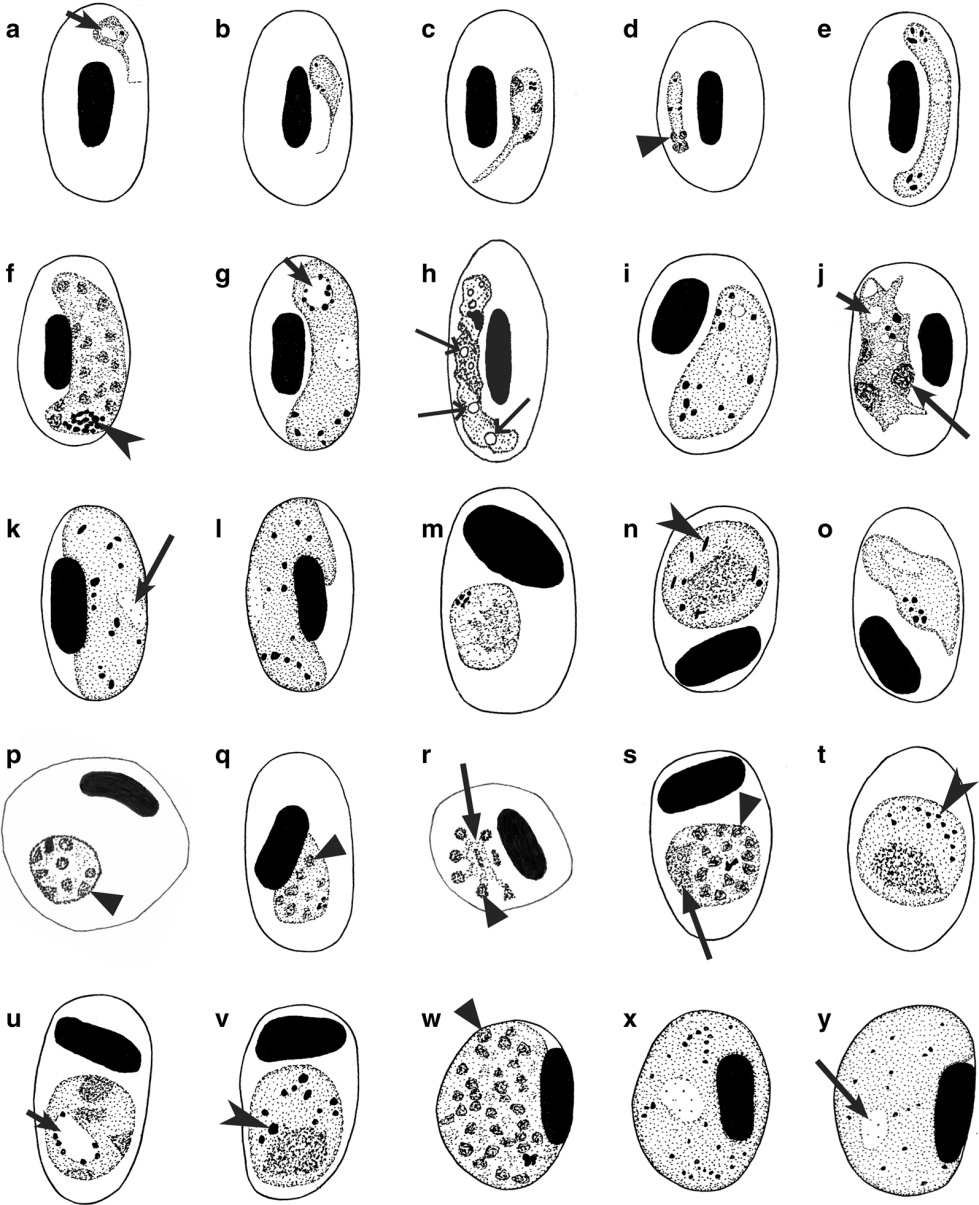
Morphological features of blood stages and their host cells of avian *Plasmodium* parasites, which are used for species identification. Young trophozoite (**a**) and gametocyte (**b**), growing erythrocytic meronts (**c**, **d**, **j**, **u**), mature erythrocytic meronts (**f**, **p**–**s**, **w**), and mature gametocytes (**e**, **g**–**i**, **k**–**o**, **t**, **v**, **x**, **y**). Note presence of long outgrowths (**a**–**c**), terminal position of nuclei in meront (**d**), slender shape of gametocyte (**e**), aggregation of pigment granules at one end of gametocyte (**f**), rod-like pigment granules (**n**), large vacuoles (**g**, **j**, **u**), refractive globules in gametocyte (**h**), oblique position of gametocytes in erythrocytes (**i**, **o**), strictly nucleophilic erythrocytic meronts (**q**), residual cytoplasm in erythrocytic meronts (**r**, **s**), rounded shape of infected erythrocytes (**p**, **w**–**y**). Triangle long arrows—residual body in mature meront. Symbols as in Figs. 1, 2, 3, 4. Explanations are given in the text

^c^Exo-erythrocytic merogony takes place in cells of the reticuloendothelial system. Erythrocytic trophozoites and growing meronts contain scanty cytoplasm (Fig. 3g). Growing erythrocytic meronts are nucleophilic. The size of fully grown erythrocytic meronts does not exceed that of the nuclei of infected erythrocytes (Fig. 3g, s). Fully grown gametocytes are roundish, oval, of irregular form, sometimes oval-elongated; their size does not exceed that of the nuclei of infected erythrocytes (Fig. 5m). Pedunculated oocysts are present. Subgenus *Bennettinia* contains only one species, *Plasmodium juxtanucleare* [81]

^d^Exo-erythrocytic merogony takes place in cells of the reticuloendothelial system. Erythrocytic trophozoites (Fig. 3d) and growing meronts (Fig. 3x) contain plentiful cytoplasm. The size of fully grown erythrocytic meronts exceeds that of the nuclei of infected erythrocytes (Figs. 2d, e; 3w, y). Fully grown gametocytes are elongated (Figs. 4c–s; 5i, k, o). Pedunculated oocysts are absent

^e^Exo-erythrocytic merogony takes place in cells of the reticuloendothelial system. Erythrocytic trophozoites (Fig. 3a, b) and growing meronts (Fig. 3e–j) contain scanty cytoplasm. The size of fully grown erythrocytic meronts does not exceed or only slightly exceeds that of the nuclei of infected erythrocytes occasionally (Fig. 3p–r). Fully grown gametocytes are elongated (Fig. 4c–r). Pedunculated oocysts are absent

When the subgenus of a malaria parasite has been identified, the next step is the species identification using the keys to species (Tables 3, 4, 5, 6).

**Table 3:** Key to the *Haemamoeba* species

Step	Features and species	
1 (16)	Roundish or oval pigment granules predominate in gametocytes (Fig. 4u–x). Elongate rod-like in form pigment granules (Fig. 5n) are absent, but single slightly elongate pigment granules might occur occasionally	
2 (22)	A residual body (Fig. 5s) is absent in mature erythrocytic meronts. Of oval-elongate form gametocytes, which are over 10 µm in length (Fig. 5i), are present	
3 (17)	Large (≥ 1 µm in diameter) vacuoles (Figs. 2g, h, 5u) are absent from growing erythrocytic meronts. Markedly vacuolated erythrocytic meronts (Fig. 2f–h) are absent	
4 (9)	Maximum number of merozoites in mature (Figs. 2e, j, 3n–r, y, 5r, s) erythrocytic meronts is ≤ 12	
5 (8)	Maturing and mature erythrocytic meronts enlarge infected erythrocytes < ½ in area in comparison to uninfected erythrocytes (compare infected and uninfected erythrocytes in Fig. 2g, h, see also Fig. 5s); numerous mature meronts adhere to erythrocyte nuclei (Fig. 2j)	
6 (7)	Merozoites locate haphazardly in mature meronts (Fig. 1c). Residuum cytoplasm (Fig. 5s) is invisible in mature meronts, and merozoites never appear to have connections to the residuum cytoplasm	
	…………………………………………… *P. subpraecox* [1, 8, 82]	
7 (6)	Nuclei locate on periphery of maturing and mature meronts (Fig. 5r). Residuum cytoplasm is visible and locates centrally in maturing meronts (Fig. 5r). Maturing merozoites have connections to the residuum cytoplasm, and these connections look like small wisps of cytoplasm extending towards merozoites (Fig. 5r)	
	…………………………………………… *P. parvulum* [51]	
8 (5)	Maturing and mature meronts enlarge infected erythrocytes over ½ in area in comparison to uninfected erythrocytes (Fig. 5p). The majority of maturing meronts are rounded in shape, they locate away from erythrocyte nuclei, which are markedly displaced toward erythrocyte envelope from earliest stages of meronts development (Fig. 5p)	
	…………………………………………… *P. caloti*^a^ [57]	
9 (4)	Maximum number of merozoites in mature erythrocytic meronts is > 12. Mature meronts and gametocytes are large (size is significantly greater than erythrocyte nuclei); they occupy > 1/2 of the cytoplasm in infected erythrocytes (Figs. 2j; 4u–x)	
10 (23)	Pigment granules in gametocytes do not tend to be clumped in a spot, which is usually located near a margin of the parasite (Fig. 4t, w). If present occasionally, such position of pigment granules does not predominate in mature gametocytes	
11 (10)	Pigment granules in mature gametocytes show markedly different patterns of position in the cytoplasm; they often are randomly scattered (Figs. 1b, 5v), but also might be variously grouped (Fig. 5t) and even aggregated in solid masses (Fig. 4u, v, x)	
12 (15)	Largest fully grown gametocytes can occupy all available cytoplasmic space in infected erythrocytes (Fig. 5x, y). Length of the largest gametocytes exceed 10 µm	
13 (14)	Development in the blood is asynchronous, with all blood stages present in circulation simultaneously. Periodicity of erythrocytic merogony is 36 h; Specific parasite of domestic chicken. Passeriform birds are resistant. In the nature, transmission does not occur outside the Oriental zoogeographical region	
	…………………………………………… *P. gallinaceum* [1, 8, 83]	
14 (15)	Development in the blood is synchronous, with not all blood stages present in circulation simultaneously. Periodicity of erythrocytic merogony is 24 h. Domestic chicken was reported to be resistant. In the nature, transmission occurs outside the Oriental zoogeographical region	
	…………………………………………… *P. coturnixi* [8, 84]	
15 (12)	Largest fully-grown gametocytes do not occupy all available cytoplasmic space in infected erythrocytes; a small non-occupied space is usually visible in infected erythrocytes (Fig. 4u–x). Length of the largest gametocytes does not exceed 10 µm. Domestic chicken is resistant. Development in the blood is asynchronous, with all blood stages (trophozoites, growing and mature meronts as well gametocytes) present in blood films simultaneously. Periodicity of erythrocytic merogony is 36 h	
	…………………………………………… *P. relictum* [8, 26, 85]	
16 (1)	Pigment granules in gametocytes are roundish, oval and elongate rod-like (Fig. 5n). Rod-like pigment granules are common and might predominate in microgametocytes (Fig. 5n), but they are less common and often do not predominate in macrogametocytes	
	…………………………………………… *P. cathemerium* [1, 8, 86]	
17 (3)	Large (≥ 1 µm in diameter) vacuoles (Figs. 2h; 5u) are common in erythrocytic meronts	
18 (21)	One or several large vacuoles, which do not exceed 2 µm in diameter, are often present in growing erythrocytic meronts. Markedly vacuolated erythrocytic meronts are common (Fig. 2f–h). Pigment granules do not gather around these vacuoles. Trophozoites lack large (> 1 µm in diameter) vacuoles. Lobulated in form gametocytes (Fig. 4x) are absent or develop only occasionally	
19 (20)	Vacuoles are absent or occur occasionally in erythrocytic trophozoites. Pigment granules in fully grown gametocytes distinctly vary in size, and small (< 0.5 µm) and medium (0.5–1.0 µm) size granules occur simultaneously (Fig. 5v). The medium-size pigment granules are common (Fig. 5v). Phanerozoites do not develop in brain of domestic canaries	
	…………………………………………… *P. giovannolai* [1, 8, 87]	
20 (19)	Vacuoles often present in erythrocytic trophozoites. Pigment granules in fully grown gametocytes are more or less similar in size, usually they are small (< 0.5 µm) (Fig. 5t). Medium-size (0.5–1.0 µm) pigment granules (Fig. 5v) might occur, but are not characteristic. Phanerozoites develop in brain of domestic canaries	
	…………………………………………… *P. matutinum* [1, 8, 66, 88]	
21 (19)	Each advanced trophozoites possess one large (> 1 µm in diameter) roundish centrally located vacuole. One large (> 2 µm in diameter) vacuole is present in growing erythrocytic meronts (Fig. 5u). Pigment granules gather around this vacuole. Lobulated in form gametocytes (Fig. 4x) are common	
	…………………………………………… *P. tejerai* [8, 50, 89]	
22 (2)	A residual body (Fig. 5s) is present in mature erythrocytic meronts. Of oval-elongate form gametocytes, which are over 10 µm in length (Fig. 5i), are present. Growing erythrocytic meronts often possess vacuoles (Fig. 2g, h)	
	…………………………………………… *P. griffithsi* [1, 8]	
23 (10)	Pigment granules in gametocytes clearly tend to be clumped in a spot, which is located near a margin of the parasite (Fig. 4t, w). Such position of pigment granules predominates in mature gametocytes. Pigment granules can be aggregated into a solid mass of pigment, which also usually locates near a margin of the parasite	
	…………………………………………… *P. lutzi* [8, 90, 91]	

^a^*Plasmodium caloti* was described from the Eurasian skylarks *Alauda arvensis* co-infected with several other *Plasmodium* species, and this races a question if all blood stages (particularly gametocytes), which were reported in the original description [57], truly belong to this parasite. However, because of (1) the marked influence on host cell (marked enlargement of infected erythrocytes and displacement of their nuclei) and (2) the relatively regular rounded form and smooth margins of mature meronts (Fig. 5p), which produce small number of merozoites < 10), this parasite is morphologically unique and can be distinguished from other *Haemamoeba* species. The original description is fragmentary [57], and re-description of this parasite is needed

**Table 4:** Key to *Giovannolaia* species

Step	Features and species	
1 (16)	Elongate meronts, which grow laterally to nuclei of infected erythrocytes (Figs. 2c–e; 3w–y), predominate	
2 (3)	Cytoplasm of gametocytes (especially macrogametocytes) is highly vacuolated (Fig. 4o). Large (> 1.5 µm in diameter) vacuoles are present in some macrogametocytes	
	………………………………………………. *P. fallax* [1, 8, 92]	
3 (2)	Cytoplasm of gametocytes is not highly vacuolated; if vacuoles are present in macrogametocytes, they are few and of small size (< 1 µm in diameter) (Fig. 4p–s)	
4 (5)	Pigment granules in the majority of erythrocytic meronts are aggregated into large (> 1.5 µm in length) clumps, which usually locate at one end of elongate meronts (Fig. 5f)	
	………………………………………………. *P. anasum* [1, 8, 93]	
5 (4)	Pigment granules in the majority of erythrocytic meronts are not aggregated into large (> 1.5 µm in length) clumps, which usually locate at one end of elongate meronts (Fig. 5f). Location of pigment granules in erythrocytic meronts is markedly variable	
6 (7)	Nuclei tend to lean to one end in the majority of growing erythrocytic meronts (Fig. 5d)	
	………………………………………………. *P. leanucleus* [8, 94]	
7 (6)	Nuclei do not tend to lean to one end in the majority of growing erythrocytic meronts (Fig. 5d). Position of nuclei in developing meronts is markedly variable (Fig. 3u, x)	
8 (11)	Average number of merozoites in mature meronts is < 12	
9 (10)	Fully grown erythrocytic meronts and gametocytes are thin slender cells, they do not displace the nuclei of infected erythrocytes and usually do not adhere to the nuclei (Fig. 5e)	
	………………………………………………. *P. gundersi* [1, 8, 95]	
10 (9)	Fully grown erythrocytic meronts (Fig. 3w) and gametocytes (Fig. 5k) are broad cells, which width is equal to the width of erythrocyte nuclei or is greater; both mature meronts and gametocytes displace the nuclei of infected erythrocytes laterally and often adhere to the nuclei (Figs. 3w; 5k)	
	………………………………………………. *P. octamerium* [8, 96]	
11 (8)	Average number of merozoites in mature meronts is ≥ 12	
12 (15)	Gametocytes and meronts grow around nuclei of erythrocytes (Figs. 2c, d; 4r, s). Fully grown erythrocytic meronts and gametocytes usually only slightly (if at all) influence infected erythrocytes and do not displace or only slightly displace nuclei of erythrocytes laterally (Figs. 2d, e; 4s). Infected erythrocytes usually do not become rounded (Fig. 5x)	
13 (14)	Fully-grown erythrocytic meronts (Fig. 2d, e) and gametocytes (Fig. 4s) markedly (often nearly completely or completely) encircle nuclei of infected erythrocytes; completely circumnuclear mature meronts and gametocytes frequently develop, but their occurrence depends of stage of parasitemia, so they might be not always seen in blood films	
	………………………………………………. *P. circumflexum* [1, 8, 97] , *P. homocircumflexum* [35]^a^	
14 (13)	Fully grown erythrocytic meronts never assume circumnuclear form (Fig. 3w, y). Gametocytes nearly completely (Fig. 4r) or completely (Fig. 4s) encircle nuclei of infected erythrocytes; completely circumnuclear mature gametocytes develop, but usually are rare. Advanced trophozoites and young meronts often possess large (> 1 µm in diameter) vacuoles (Fig. 5j)	
	………………………………………………. *P. lophurae* [1, 8, 98]	
15 (12)	Gametocytes and meronts start to grow around nuclei of erythrocytes However, fully grown meronts markedly displace nuclei of erythrocytes and assume various irregular forms; they often roundish or close to roundish in shape (Fig. 5w), markedly displace the nuclei of infected erythrocytes and can occupy all available cytoplasmic space in the erythrocytes (Fig. 5w). Fully grown gametocytes markedly deform infected erythrocytes, which become rounded (Fig. 5x, y)	
	………………………………………………. *P. gabaldoni* [8, 99]	
16 (1)	Elongate erythrocytic meronts, which grow laterally to nuclei of infected erythrocytes (Figs. 2c–e; 3w–y), are absent or appear only occasionally; they never predominate. The majority of fully grown meronts are of roundish, oval or irregular form; they do not take or take only occasionally the lateral position to nuclei of erythrocytes (Fig. 5q–s)	
17 (24)	Large (> 1.5 µm in diameter) vacuoles (Fig. 5g) absent from gametocytes. If small vacuoles are present in gametocytes, pigment granules do not gather around vacuoles	
18 (25)	Fully grown gametocytes do not tend to lie obliquely in infected erythrocytes (Fig. 5i, o), and they do not displace the nuclei towards one pole of the erythrocytes	
19 (26)	Growing erythrocytic meronts do not produce long (> 2 µm in length) tail-like or finger-like outgrowths (Fig. 5c)	
20 (21)	Erythrocytic meronts take a polar or subpolar position in infected erythrocytes, and their influence on infected erythrocytes is usually not pronounced (Fig. 3r)	
	………………………………………………. *P. polare* [1, 8, 100]	
21 (20)	Erythrocytic meronts can be seen anywhere in infected erythrocytes including a lateral, subpolar and polar position. If meronts take a polar or subpolar position in the erythrocytes, they markedly influence the host cells causing their deformation and (or) displacement of their nuclei	
22 (23)	Maximum number of merozoites in mature meronts > 10. Size of pigment granules in macro- and microgametocytes is clearly different	
	………………………………………………. *P. pinottii* [1, 8, 101]	
23 (22)	Maximum number of merozoites in mature meronts < 10. Size of pigment granules in macro- and microgametocytes is similar	
	………………………………………………. *P. garnhami* [1, 8, 102]	
24 (17)	Large (> 1.5 µm in diameter) vacuoles (Fig. 5g) develop in many macrogametocytes. Pigment granules often gather around these vacuoles	
	………………………………………………. *P. formosanum* [1, 8, 103]	
25 (18)	Fully grown gametocytes tend to lie obliquely in infected erythrocytes, and they displace the nuclei towards one pole of the erythrocytes (Fig. 5o)	
	………………………………………………. *P. durae* [1, 8, 104]	
26 (19)	Growing erythrocytic meronts often produce long (> 2 µm in length) tail-like or finger-like outgrowths (Fig. 5c)	
27 (28)	Nuclei in mature erythrocytic meronts are usually arranged as fans (Fig. 3v), rosettes (Fig. 5r), or more or less pronounced rows (Fig. 3w). Infected erythrocytes with segmented mature meronts are often rounded (Fig. 5r). Fully grown gametocytes do not fill erythrocytes up to their poles (Fig. 4h)	
	………………………………………………. *P. pedioecetae* [1, 8, 105, 106]	
28 (27)	Nuclei in mature erythrocytic meronts are usually located randomly (Fig. 3q) and they only occasionally can be arranged as rosettes. Infected erythrocytes with segmented mature meronts are not rounded (Fig. 3w). Fully grown gametocytes fill erythrocytes up to their poles (Fig. 5k)	
	………………………………………………. *P. hegneri* [8, 93]	

^a^Based on available information, *P. circumflexum* and *P. homocircumflexum* are cryptic species, which cannot be distinguished using morphological features of their blood stages [35]. Cytochrome *b* sequences can be used to distinguish these infections (see Table 7)

**Table 5:** Key to the *Novyella* species

Step	Features and species	
1 (19)	Maximum number of merozoites in erythrocytic meronts > 4	
2 (41)	Maturing erythrocytic meronts, which displace host-cell nuclei, assume a fan-like shape and possess elongate nuclei (Fig. 3v), are absent	
3 (26)	Erythrocytic meronts, which lie free in the cytoplasm of host cell and do not touch the nuclei of infected erythrocytes (Fig. 3e–l, o), are present	
4 (32)	Trophozoites and binuclear meronts (Fig. 3e, f) do not produce clearly defined long outgrowths (Fig. 5a); if ameboid outgrowths are present, they do not exceed the main body of the trophozoites in length	
5 (42)	Ends of growing macrogametocytes are similar in width (Fig. 4c–e, g–r)	
6 (9)	Number of merozoites in mature erythrocytic meronts is relatively stable. Over 90% of the mature meronts contain 6 merozoites	
7 (8)	Macrogametocyte nuclei are terminal in position (Fig. 4g). Refractive globules (Fig. 3h) are present in erythrocytic meronts	
	……………………………………………. *P. parahexamerium* [55]	
8 (7)	Macrogametocyte nuclei are central or subcentral in position (Fig. 4o). Refractive globules are absent from erythrocytic meronts (Fig. 3p)	
	……………………………………………. *P. hexamerium* [1, 8, 55, 107]	
9 (6)	Number of merozoites in mature erythrocytic meronts is variable	
10 (37)	Growing and mature meronts assume various positions to the erythrocyte nuclei; they can be found in polar, sub-polar and lateral position in relation to the host cell nuclei	
11 (38)	Binuclear erythrocytic meronts do not possess large (of size, which is similar to nuclei of the meronts), centrally located vacuoles (Fig. 3e). Macro- and microgametocytes are of similar shape, they assume similar positions in erythrocytes (Fig. 4g, h)	
12 (39)	Gametocytes do not possess refractive globules	
13 (16)	Erythrocytic meronts possess globules in natural infections (Fig. 3f, h–j)	
14 (15)	The majority of trophozoites as well as developing and mature erythrocytic meronts possess one of circular shape, prominent (on average 0.5 µm^2^ in area) pigment granule (Fig. 3q). Fan-like in shape mature meronts predominate	
	……………………………………………. *P. unalis* [49]	
15 (14)	The majority of trophozoites, developing and mature erythrocytic meronts possess 1–4 (usually 2–3) small (< 0.5 µm^2^ in area), of different size pigment granules (Fig. 3c, h). Fan-like in shape mature meronts (Fig. 3o) are uncommon	
	……………………………………………. *P. vaughani* [1, 8, 49, 55, 108]	
16 (13)	Erythrocytic meronts do not possess globules in natural infections (Fig. 3g, n, o, r, s)	
17 (18)	Fan-like mature meronts containing 7–8 merozoites are common (Fig. 3o); pigment granules in gametocytes are clumped together into a prominent group, which is predominantly of terminal position in the gametocytes (Fig. 4e, h)	
	……………………………………………. *P. ashfordi* [71]	
18 (17)	Fan-like mature meronts containing 7–8 merozoites are absent; pigment granules in gametocytes are scattered or clumped, but position of these clumps is irregular (never predominantly terminal) in the gametocytes	
	……………………………………………. *P. forresteri* [8, 109]	
19 (1)	Maximum number of merozoites in erythrocytic meronts is 4	
20 (21)	Erythrocytic meronts do not possess globules in natural infections (Fig. 3g)	
	……………………………………………. *P. bertii* [8, 110]	
21 (20)	Erythrocytic meronts possess globules in natural infections (Fig. 3f, h–l)	
22 (23)	One small (< 0.5 µm in diameter) refractive globule present in the majority of meronts (Fig. 3f, h, j). Blue non-refractive globules (Fig. 3k, l) are absent from meronts. The cytoplasm in gametocytes is more or less homogenous, but never is granular-like (Fig. 4c) or globular-like (Fig. 4l, m) in appearance	
	……………………………………………. *P. rouxi* [1, 8, 52, 111]	
23 (22)	Refractive globules (Fig. 3f, h–j) are absent from meronts. One blue non-refractive globule present in each advanced trophozoite (Fig. 3b), growing and mature meront (Fig. 3k, l). The cytoplasm in gametocytes is granular-like (Fig. 4c) or globular-like in appearance (Fig. 4l, m)	
24 (25)	One large (size similar to parasite nuclei or greater) blue non-refractive globule present in each advanced trophozoite (Fig. 3b), growing and mature meront (Fig. 3l). The cytoplasm in macro- and microgametocytes is markedly globular-like in appearance (Fig. 4l, m). Average number of pigment granules in macro- and microgametocytes is close to 10	
	……………………………………………. *P. megaglobularis* [52]	
25 (24)	One small (size smaller than parasite nuclei) blue non-refractive globule present in each advanced trophozoite, growing and mature meront (Fig. 3k). The cytoplasm in gametocytes is markedly granular in appearance, which is better visible in macrogametocytes (Fig. 4c); globular-like appearance of the cytoplasm (Fig. 4l, m) is not characteristic. Average number of pigment granules in macro- and microgametocytes is close to 5	
	……………………………………………. *P. globularis* [52]	
26 (3)	Erythrocytic meronts, which lie free in the cytoplasm of host cell and do not touch the nuclei of infected erythrocytes (Fig. 3e–l, o), are absent. Erythrocytic meronts are strictly nucleophilic (Figs. 3n, s; 5q)	
27 (40)	Both meronts (Fig. 3n, p, s, t) and gametocytes (Fig. 4a, d) are strictly nucleophilic	
28 (29)	Large (≥ 1 mµ in length) pigment granules are present in gametocytes (Fig. 4d); both mature gametocytes (Fig. 4d) and meronts (Fig. 3n, t) do not displace nuclei of erythrocytes	
	……………………………………………. *P. delichoni* [72]	
29 (30)	Large (≥ 1 mµ) pigment granules (Fig. 4d) are absent from gametocytes; mature meronts (Fig. 5q) or mature gametocytes (Fig. 5k) displace nuclei of erythrocytes	
30 (31)	Fully-grown gametocytes do not fill erythrocytes up to their poles (Fig. 4h). Fully grown meronts displace nuclei of erythrocytes (Fig. 5q). Fully-grown gametocytes do not displace the nuclei of infected erythrocytes (Fig. 4h)	
	……………………………………………. *P. nucleophilum* [1, 8, 112–114]	
31 (30)	Fully grown gametocytes fill erythrocytes up to their poles (Fig. 5k). Fully grown erythrocytic meronts do not displace or only slightly displace the nuclei of infected erythrocytes (Fig. 3t). Fully-grown gametocytes markedly displace the nuclei of infected erythrocytes laterally (Fig. 5k)	
	……………………………………………. *P. paranucleophilum* [8, 113]	
32 (4)	Trophozoites and (or) binuclear meronts often produce clearly defined long outgrowths (Fig. 5a); the outgrowths exceed the main body of the trophozoites in length	
33 (34)	One or two refractive globules present in advanced trophozoites and developing and mature meronts. Each globule has a clear rim at its periphery (Fig. 3i); macrogametocytes are markedly vacuolated in appearance (Fig. 4n)	
	……………………………………………. *P. multivacuolaris* [55]	
34 (33)	Globules are absent from trophozoites and meronts, but vacuoles might be present	
35 (36)	A large (> 1 µm in length) vacuole is present in trophozoites (Fig. 5a). Number of merozoites in erythrocytic meronts is relatively stable; approximately 95% of the meronts contain five merozoites. Pigment granules in gametocytes either randomly scattered throughout the cytoplasm or clumped into several small groups	
	……………………………………………. *P. kempi* [8, 115]	
36 (35)	A large (> 1 µm in length) vacuole (Fig. 5a) is absent from trophozoites, but a small (< 1 µm in diameter) vacuole present occasionally. Number of merozoites in erythrocytic meronts is variable, but most often is equal to 8. Pigment granules in gametocytes frequently are clumped in a focus near one end of the gametocytes (Fig. 4h)	
	……………………………………………. *P. columbae* [1, 8, 116]	
37 (10)	Growing and mature meronts are strictly of polar or subpolar position to the erythrocyte nuclei, and they usually do not adhere to the nuclei (Fig. 3q). Refractive globules present in grooving meronts, but often are invisible in mature meronts	
	……………………………………………. *P. homopolare* [58]	
38 (11)	Binuclear erythrocytic meronts often possess one large (size similar to nuclei of the meronts), centrally located vacuole (Fig. 3e). In binuclear meronts, nuclei locate asymmetrically in relation to the vacuole (Fig. 3e). Shape of macro- and microgametocytes is different: microgametocytes are elongate (Fig. 4j), but macrogametocytes are not, and they are more or less roundish, lobulated or irregular in form parasites (Fig. 4b). Macrogametocytes usually take polar or subpolar position to nuclei of erythrocytes (Fig. 4b), and microgametocytes locate laterally to the nuclei of erythrocytes (Fig. 4j). Trophozoites possess one clear vacuole (Fig. 3a), which maintain in fully grown meronts (Fig. 3m)	
	……………………………………………. *P. lucens* [55]	
39 (12)	Gametocytes possess refractive globules (Fig. 5h). Refractive circular globules present in macro- and microgametocytes	
	……………………………………………. *P. accipiteris* [53]	
40 (27)	Meronts are strictly nucleophilic (Fig. 3p), but gametocytes are not (Fig. 4r). The majority of gametocytes do not adhere to nuclei of infected erythrocytes. Mature gametocytes markedly enclose nuclei of erythrocytes by their ends (Fig. 4r)	
	……………………………………………. *P. homonucleophilum* [117]	
41 (2)	Maturing erythrocytic meronts, which displace host-cell nuclei, assume fan-like shape and possess elongate nuclei (Fig. 3v), are present. Number of nuclei in maturing fan-like meronts is about 10–12	
	……………………………………………. *P. valkiunasi*^a^ [56]	
42 (5)	Ends of growing macrogametocytes are markedly different in width	
	……………………………………………. *P. dissanaikei* [1, 8, 118]	

^a^*Plasmodium valkiunasi* was described from Eurasian magpies *Pica pica* co-infected with several other *Plasmodium* species, and this races a question if all blood stages (particularly gametocytes), which were reported in the original description [56], truly belong to this species. However, this parasite is morphologically unique and can be distinguished from other *Novyella* species because of unique shape of its maturing meronts (Fig. 3v), which are large, develop in mature erythrocytes, have a regular fan-like form and possess numerous (about 12) peripherally located elongate nuclei. The original description is fragmentary [56], and re-description of this parasite is needed

**Table 6:** Key to *Huffia* species

Step	Features and species	
1 (2)	Development and maturation of gametocytes occurs in various immature red blood cells, including erythroblasts (Fig. 4y). The outline of nuclei in growing erythrocytic meronts (Fig. 2m, n) as well as growing and mature gametocytes (Fig. 4y) is smooth, and boundaries between nuclei and the cytoplasm are strictly distinct (Figs. 2m, n; 4y)	
	………………………………………………… *P. polymorphum* [59]	
2 (1)	Development and maturation of gametocytes occurs only in mature or nearly mature red blood cells (Fig. 4p, q); gametocytes do not develop in erythroblasts. The outline of nuclei in growing erythrocytic meronts (Fig. 2l, o, p) as well as growing and mature gametocytes (Fig. 4p, q) is markedly variable, predominantly not smooth, and boundaries between the nuclei and the cytoplasm are often poorly distinct (Figs. 2o, p; 4p, q), particularly in the growing parasites	
3 (6)	In peripheral blood, trophozoites and erythrocytic meronts develop mainly in young red blood cells. Maximum number of merozoites in erythrocytic meronts is less than 20	
4 (5)	Elongated erythrocytic merozoites are present (Fig. 2k). Fully grown gametocytes are slender; they do not displace or only slightly displace the nuclei of infected erythrocytes laterally (Fig. 4p, q). Maximum width of fully grown gametocytes is equal or less than the width of nuclei of host cells (Fig. 4p, q)	
	………………………………………………… *P. elongatum* [1, 8, 23, 119, 120]	
5 (4)	Elongated erythrocytic merozoites (Fig. 2k) are absent. Fully grown gametocytes are broad; they markedly displace the nuclei of infected erythrocytes laterally and can fill the poles of infected erythrocytes completely (Fig. 5l). Maximum width of fully grown gametocytes is greater than the width of nuclei of host cells (Fig. 5l)	
	………………………………………………… *P. hermani* [8, 120, 121]	
6 (3)	In peripheral blood, trophozoites and erythrocytic meronts develop mainly in mature red blood cells. Maximum number of merozoites in erythrocytic meronts is greater than 20	
	………………………………………………… *P. huffi* [1, 8, 122]	

## Discussion

There are three main groups of obstacles, which a researcher usually faces during morphological identification of malaria parasites using microscopic examination of blood samples collected in the field. First, the quality of microscopic preparations is essential for correct parasite identification, but often is insufficient due to thick blood films or artefacts of their drying, fixation, staining or storage. This precludes visualization of some important features for species identification. It is essential to master these simple methods of traditional parasitology before sample collection, and this can be readily achieved in each laboratory using available protocols [1, 8, 63, 64]. Second, *Plasmodium* species parasitaemia is often light in natural infections in the wild. In other words, malaria parasites might be reported in blood films, but not all stages, which are needed for parasite species identification, are present. This might limit the use of the keys. Sampling of large number of birds (20–30 individuals) belonging to the same species at a study site is often helpful to detect relatively high parasitaemia of the same pathogen and to access the full range of blood stages allowing parasite species identification. Third, co-infections of *Plasmodium* species might occur, and requires some experience to distinguish between different pathogens [45, 48, 56]. These obstacles strengthen the need for the development of molecular characterization in avian malaria diagnostics, which is still only available for 44% of described parasite species, whose validity is obvious (Table 7). This is particularly timely for itemizing *Plasmodium* species phylogenies, which currently are based mainly on mitochondrial *cytb* gene sequences in avian malariology [5, 7, 23, 29, 33].

**Table 7:** Mitochondrial cytochrome *b* sequences, which have been developed for molecular detection and identification (barcoding) of avian *Plasmodium* parasites

Parasite subgenus and species	GenBank accession and lineage code (in parentheses)^a^	References^b^
*Haemamoeba*		
*P. caloti*	Not available	Not available
*P. cathemerium*	AY377128 (pSEIAUR01)	[123]
*P. coturnixi*	Not available	Not available
*P. gallinaceum*	AY099029 (pGALLUS01)	[124]
*P. giovannolai*	Not available	Not available
*P. griffithsi*	Not available	Not available
*P. lutzi*	KC138226 (pTFUS05)	[91]
*P. matutinum*	KY287235 (pLINN1)	[66]
*P. parvulum*	Not available	Not available
*P. subpraecox*	Not available	Not available
*P. relictum*	AF495571 (pSGS1), AY831748 (pGRW11), AY099041 (pGRW4), KC342644 (pLZFUS01), MG724747 (pPHCOL01)	[67, 71, 117]
*P. tejerai*	JX272844 (pSPMAG01)	[50]
*Giovannolaia*		
*P. anasum*	Not available	Not available
*P. circumflexum*	AF495576 (pTURDUS1)	[67]
*P. durae*	Not available	Not available
*P. fallax*	Not available	Not available
*P. formosanum*	Not available	Not available
*P. gabaldoni*	Not available	Not available
*P. garnhami*	Not available	Not available
*P. gundersi*	Not available	Not available
*P. hegneri*	Not available	Not available
*P. homocircumflexum*	KC884250 (pCOLL4)	[35]
*P. leanucleus*	Not available	Not available
*P. lophurae*	Not available	Not available
*P. polare*	Not available	Not available
*P. octamerium*	Not available	Not available
*P. pedioecetae*	Not available	Not available
*P. pinottii*	Not available	Not available
*Novyella*		
*P. accipiteris*	Not available	Not available
*P. ashfordi*	AF254962 (pGRW2)	[71]
*P. bertii*	Not available	Not available
*P. columbae*	Not available	Not available
*P. delichoni*	KU529943 (pCOLL6)	[72]
*P. dissanaikei*	Not available	Not available
*P. forresteri*	Not available	Not available
*P. globularis*	EU770151 (pANLA1)	[52]
*P. hexamerium*	Not available	Not available
*P. homonucleophilum*	KC342643 (pSW2)	[117]
*P. homopolare*	KJ482708 (pSOSP CA 3P)	[58]
*P. kempi*	Not available	Not available
*P. lucens*	FJ389156 (pCYOL2)	[55]
*P. megaglobularis*	EU770152 (pCYOL1)	[52]
*P. multivacuolaris*	FJ389157 (pANLA2)	[55]
*P. nucleophilum*	JX467689 (pEG01)	[114]
*P. parahexamerium*	FJ389155 (pALDI1)	[55]
*P. paranucleophilum*	Not available	Not available
*P. rouxi*	HM146901 (pPADOM16)	[68]
*P. unalis*	KC771247 (pTFUS06)	[49]
*P. valkiunasi*	Not available	Not available
*P. vaughani*	DQ847271 (pSYAT05)	[22]
*Bennettinia*		
*P. juxtanucleare*	AB250415 (pGALLUS02)	[125]
*Huffia*		
*P. elongatum*	DQ368381 (pGRW6); KT282462 (pERIRUB01)	[23, 120]
*P. hermani*	Not available	Not available
*P. huffi*	Not available	Not available
*P. polymorphum*	Not available	Not available

^a^Only DNA sequences, for which parasite species identity was supported by morphological analysis are included in this table

^b^References of articles containing discussion of molecular characterization and morphological features of parasite species

Molecular markers are sensitive for distinguishing different parasite species and their lineages, and they are essential for identification of cryptic *Plasmodium* species [35]. Molecular characterization is best developed for *Novyella* parasites (molecular markers are available for 59% of described species of this subgenus), and is weakest for *Giovannolaia* parasites (only two species or 12.5% of this subgenus have been characterized molecularly). Lack of molecular markers for many described malaria pathogens [51, 53, 54, 56, 57, 59, 65] precludes biodiversity research on *Plasmodium* species and recognition of new malaria pathogens, for whose detection, detailed comparison with already described and genetically characterized parasites is needed. The development of molecular markers for diagnosis of disease agents is an important task of current avian malariology (Table 7).

This study shows that 55 described species of avian malaria parasites can be readily distinguished (Tables 3, 4, 5, 6, 7). Among them, 12, 16, 22, 4 and 1 species belong to subgenera *Haemamoeba, Giovannolaia, Novyella, Huffia* and *Bennettinia*, respectively. The great majority of described avian *Plasmodium* species were reported only in birds that live in tropical and subtropical countries or in Holarctic migrants wintering in the same regions, indicating that transmission of these pathogens occurs mainly in countries with warm climates. Those malaria parasites, which have adapted for transmission globally and have become cosmopolitan, are exceptions. Among these, *Plasmodium relictum, Plasmodium elongatum, Plasmodium circumflexum, Plasmodium matutinum* and *Plasmodium vaughani* should be mentioned first of all [6, 8, 21, 23, 66–70]. These are invasive infections, which are often virulent in non-adapted hosts, and they are worth particular attention in bird health.

Among described avian *Plasmodium* parasites, species of *Novyella* are particularly diverse (Table 5). They represent approximately 40% of all described avian malaria pathogens, and 78% of *Plasmodium* species, which were discovered during past 15 years. *Novyella* parasites are mainly pathogens of birds in countries of tropical and subtropical regions (Table 5). The Holarctic migrating birds gain *Novyella* infections in their wintering grounds and transport them to their breeding grounds where they are normally not transmitted [8, 71–73]. Factors preventing spread of *Novyella* infections globally are unclear. *Novyella* species are the most poorly studied group of avian malaria pathogens, with nearly no information available about exo-erythrocytic development, virulence, sporogony and vectors for the great majority [1, 4, 8, 72]. A few *Novyella* parasites (*P. vaughani, Plasmodium rouxi, Plasmodium homopolare*) are actively transmitted in countries with temperate climates, but they are absent or of low prevalence in areas with cold climates located close to the Polar Circles [1, 8, 18, 19, 58, 68].

Limited available experimental information indicates that some *Novyella* species (*P. ashfordi, P. rouxi*) may cause severe and even lethal malaria in some birds due to blood pathology [1, 8, 74, 75], but the complete mechanism of their pathogenicity remains unresolved, mainly due to lack of information about exo-erythrocytic development [72]. Investigation of life cycles and virulence of infections caused by *Novyella* species is an important task in current avian malaria research.

Many species of *Plasmodium* inhabit numerous species of birds and use mosquitoes of different genera for transmission [1, 8, 9, 11]. Within this spectrum of hosts and vectors, the same parasite species might exhibit diverse morphological forms and strain varieties. Because of these morphological variants, it has been conventional in old avian malaria research (approximately between 1927 and 1995) that any new *Plasmodium* species description should only be accepted if supported by a comprehensive package of taxonomic features, which not only included the full range of blood stages, but also data on the vertebrate host specificity, periodicity of erythrocytic merogony, tissue merogony, vectors and patterns of sporogonic development. It is not surprising that recent molecular studies supported the validity of the old *Plasmodium* species descriptions, which were detailed and precise (Table 7). Application of molecular diagnostic tools in studies of avian haemosporidian parasites [29, 69, 76, 77] opened new opportunities to distinguish haemosporidian parasites based on their unique DNA sequences. This stimulated biodiversity research of wildlife *Plasmodium* parasites, particularly because the molecular characterization, which was done in parallel with morphological description of blood stages, made each parasite species detection readily repeatable at all stages of life cycle (Table 7).

A list of synonymous names of avian *Plasmodium* species and the justification of the nomenclature status of these names are given in Table 8. The majority of these parasite descriptions are insufficiently complete and were not accompanied with molecular characterization. Due to the huge genetic diversity of avian malaria pathogens and numerous genetic lineages reported in birds, some of these names might be validated in the future, and they represent a reserve for future taxonomic work. However, available descriptions of these parasites do not provide sufficient information to readily distinguish them from parasites, whose validity is well established (Tables 3, 4, 5, 6). For clearness of scientific texts, it is preferable to avoid use of the synonymous names before additional data on their validity are available. Reports of parasite lineages and GenBank accessions of their DNA sequences in publications would be helpful to specify *Plasmodium* species identity in the future.

**Table 8:** List of synonyms of *Plasmodium* species of birds

Synonymous name and references of original description	Valid name^a^
*Plasmodium alaudae* [126]	*P. relictum* (partim)
*P. alloelongatum* [53]^b^	*P. elongatum*
*P. bioccai* [56]^c^	*P. relictum*
*P. biziurae* [127]	*P. relictum*
*P. capistrani* [128]	*P. relictum*
*P. centropi* [129]	*P. cathemerium* (partim)
*P. chloropsidis* [130]	*P. relictum*
*P. coluzzii* [57]^c^	*P. relictum*
*P. dorsti* [56]^c^	*P. relictum*
*P. ginsburgi* [57]^c^	*P. relictum*
*P. heroni* [131]	*P. circumflexum*
*P. huffi* [122]	*P. nucleophilum*
*P. japonicum* [132]	*P. juxtanucleare*
*P. metastaticum* [133]	*P. gallinaceum*
*P. grassii* [134]	*P. relictum*
*P. inconstans* [86]	*P. relictum*
*P. maior* [135]	*P. relictum*
*P. merulae* [136]	*P. vaughani*
*P. mohammedi* [54]^d^	*P. rouxi*
*P. muniae* [137]	*P. relictum*
*P. majoris* [138]	*P. relictum* (partim)
*P. oti* [139]	*P. hexamerium*
*P. passeris* [140]	*P. relictum*
*P. paddae* [141]	*P. relictum*
*P. pericrocoti* [142]	*P. relictum*
*P. ploceii* [142]	*P. relictum*
*P. relictum quentini* [57]^c^	*P. relictum*
*P. spheniscidae* [143]	*P. relictum*
*P. tumbayaensis* [144]	*P. vaughani*
*P. tenuis* [145]	*P. vaughani*
*Plasmodium wasielewskii* [146]	*P. subpraecox*

^a^*Plasmodium* species synonymous names published before 2000 were justified in [8]

^b^According to the original description [53], *P. alloelongatum* is similar to *P. elongatum*, but differs from the latter species mainly due to two characters: (1) the erythrocytic meront progeny is limited to 6 (predominantly 6–12 in *P. elongatum*), and (2) the undulating or rugged outlines and tapering gametocyte ends, which might extend into a distal spine or filaments. The irregularity of gametocyte shape (Fig. 4p) and presence of ameboid outgrowth (Fig. 4q) has been reported and illustrated in *P. elongatum* (the closely related lineages pGRW6 and pERIRUB01), but have rarely pointed out in descriptions of this parasite [1, 23, 120]. Furthermore, the ameboid outgrowths in gametocytes were seen in the neohapantotype of *P. elongatum* (blood slide no. 216, the Natural History Museum, London). The number of nuclei in mature erythrocytic meronts is variable in *P. elongatum* during development in different host cells and avian hosts, and it is often ≤ 6 [1, 23, 120]. *Plasmodium elongatum* has been characterized molecularly (Table 7), and it has been reported in numerous bird species belonging to different orders both by microscopic examination of blood films and PCR-based testing, including species of Accipitriformes and Falconiformes [21]. Based on available information, *Plasmodium alloelongatum* cannot be distinguished is considered as a synonym of *P. elongatum*

^c^Observation of blood stages in various bird species experimentally infected with single infections of *Plasmodium relictum* lineages pSGS1 and pGRW11, which are closely related and widespread in Europe, show that main reported *P. bioccai, P. coluzzii, P. dorsti, P. ginsburgi, P. relictum quentini* blood stages (meronts and gametocytes) are present in these parasite lineages [67, 71, 117]. These parasites were described in co-infection with *Haemamoeba* parasites, including *P. relictum*, and description of blood stages were fragmentary [56, 57]. Blood stages of all these parasites do not have unique characters, which could help to distinguish them from *P. relictum*. *Plasmodium bioccai, P. coluzzii, P. dorsti, P. ginsburgi, P. relictum quentini* are considered as synonyms of *P. relictum*

^d^Synonymous status of *P. mohammedi* was specified in Table 9 (see the footnote “f”)

A list of the *Plasmodium* species names of unknown taxonomic position (*incertae sedis*) and also the names of species of doubtful identity, which require further investigation (*species inquirenda*), is given in Table 9. All these parasite descriptions are insufficiently complete and were not accompanied with molecular characterization. Taxonomic status of the majority of these names was justified in [8]. Twenty names of *Plasmodium* parasites were added to this list and their taxonomic status was explained (Table 9). The majority of these parasite descriptions are based on preparations with co-infections of several *Plasmodium* parasites belonging to same and (or) different genera. This raises a question if all blood stages reported in the original descriptions truly belong to corresponding species.

**Table 9:** List of species names of bird malaria parasites belonging to the categories of *nomen nudum*, *nomen dubium*, *species inquirenda* and *incertae sedis*

Name and references	Status^a^
*Plasmodium alaudae* [57, 126]^b^	*Species inquirenda*
*P. arachnidi* [147]	*Species inquirenda*
*P. bambusicolai* [148]	*Species inquirenda*
*P. beaucournui* [56]^b^	*Species inquirenda*
*P. bigueti* [65]^b^	*Species inquirenda*
*P. buteonis* [53]^c^	*Species inquirenda*
*P. coggeshalli* [149]^b^	*Species inquirenda*
*P. conturnixae* [150]	*Nomen nudum*
*P. corradettii* [151]	*Nomen dubium*
*P. danilewskyi* [152, 153]	*Incertae sedis*
*Plasmodium dherteae* [56]^b^	*Species inquirenda*
*P. gallinulae* [130]	*Incertae sedis*
*P. gambeli* [3]	*Nomen nudum*
*P. ghadiriani* [56]^b^	*Species inquirenda*
*P. golvani* [56]^b^	*Species inquirenda*
*P. herodiadis* [130]	*Species inquirenda*
*P. holti* [154]	*Nomen nudum*
*P. jiangi* [155]	*Species inquirenda*
*P. jeanriouxi* [57]^b^	*Species inquirenda*
*P. lagopi* [156]	*Species inquirenda*
*P. lairdi* [157]	*Nomen nudum*
*P. lenoblei* [56]^b^	*Species inquirenda*
*P. malariae raupachi* [158]	*Incertae sedis*
*P. manwelli* [159]	*Nomen nudum*
*P. ninoxi* [160]^d^	*Species inquirenda*
*P. noctuae* [3, 126]	*Species inquirenda*
*P. pachysomum* [54]^e^	*Species inquirenda*
*P. papernai* [149]^b^	*Species inquirenda*
*Plasmodium pfefferi* [54]^e^	*Species inquirenda*
*P. praecox* [152]	*Nomen nudum*
*P. reniai* [57]^b^	*Species inquirenda*
*P. rousseloti* [161]	*Species inquirenda*
*P. rouxi,* as published in [54]^f^	*Species inquirenda* (probably a new *Plasmodium* species)
*P. sergentorum* [54]^e^	*Species inquirenda*
*P. snounoui* [56]^b^	*Species inquirenda*
*P. spartani* [162]	*Nomen nudum*
*P. stellatum* [54]^e^	*Species inquirenda*
*P. struthionis* [163]	*Incertae sedis*
*P. tranieri* [56]^b^	*Species inquirenda*
*P. venkataramiahii* [164]	*Nomen nudum*

^a^Nomenclature status of the species names published before 2000 was justified in [8]

^b^*Plasmodium beaucournui, P. bigueti, P. coggeshalli, P. dherteae, P. ghadiriani, P. golvani, P. jeanriouxi, P. lenoblei, P. papernai, P. reniai, P. snounoui, P. tranieri* were named and described, and *P. alaudae* was re-described from individual birds co-infected with parasites belonging to subgenera *Haemamoeba, Giovannolaia* and *Novyella* [56, 57, 65, 149]. The authors of the original descriptions have grouped the blood stages visible in blood films and attributed them to different species provisionally, which is particularly obvious in case of parasites with elongate gametocytes. This makes species description and validation of parasite names questionable. Only single cells (erythrocytic meronts) were selected as holotypes in these parasite descriptions. However, due to morphological variation of blood stages of *Plasmodium* and presence of parasites at different stages of growth in each blood film, such methodology of designation of the type material can work only in case of exceptionally distinctive cell characters, which is not the case in all these parasite descriptions, particularly belonging to subgenus *Haemamoeba*. Molecular characterization of all these parasites is unavailable. It is clear from the original descriptions, that many individual birds were infected by representatives of several subgenera. However, the reported blood stages were selected and attributed to certain species without providing convincing explanations, making identifications difficult or even impossible based on available information. Co-infections of *Plasmodium* parasites belonging to different subgenera are common in wildlife, and the described cases of co-infections with several malaria parasites are not unpredictable [45]. However, description of new species from such co-infections hardly possible if the unique morphological characters of blood stages are absent, which is the case with *P. beaucournui, P. bigueti, P. coggeshalli, P. dherteae, P. ghadiriani, P. golvani, P. jeanriouxi, P. lenoblei, P. papernai, P. reniai, P. snounoui, P. tranieri* and also in re-description of *P. alaudae*. These parasites are considered as *species inquirenda*. Recent molecular studies provided molecular markers for distinguishing blood stages of *Plasmodium* species (Table 7). Examination of blood films from experimental infections shows variations in morphological characters of same parasite lineages in different avian hosts, calling for careful application of minor differences in blood stage morphology in avian malaria parasite taxonomy, particularly during co-infections

^c^Based on available information [53], *P. buteonis* cannot be distinguished from *P. circumflexum* and other similar parasites of *Giovannolaia* (*Plasmodium gabaldoni, Plasmodium homocircumflexum*). The main feature, which has been noted to distinguish *P. buteonis* from *P. circumflexum* in the original description [53], is the presence up to 36 nuclei in mature erythrocytic meronts of the former. *Plasmodium circumflexum* produce less number of nuclei in mature meronts. However, the description of *P. buteonis* is based on high parasitemia (6.6%), with numerous multiple infections of the same erythrocytes, so it is difficult to rule out that 2 mature meronts were present in same cell in case of so great number of merozoites. Additionally, parasite morphology often changes during high parasitemia, so such samples should be carefully used in taxonomical descriptions. *Plasmodium buteonis* might be a valid name, but more research is needed to prove its validity. Molecular characterization of this parasite is absent, but is essential to solve the question about its validity

^d^*Plasmodium ninoxi* was described from owl *Ninox scutulata* in co-infection with *Haemoproteus* sp. [160]. Only one erythrocyte with 2 binuclear growing meronts was detected; no other data about merogony in the blood were provided. *Plasmodium ninoxi* gametocytes were reported to be rounded. Based on available information, it seems that infected blood was exposed to air, which stimulated rounding-up of haemoproteid gametocytes [8], which were attributed to *P. ninoxi*. DNA sequence was provided (AY099035.1), and it belongs to *Plasmodium* sp. *Plasmodium ninoxi* description is incomplete. Re-description is needed, and it is possible due to available sequence information. The most similar *cytb* sequence belong to *P. gallinaceum, P. relictum* and *P. circumflexum*

^e^Descriptions of *P. pachysomum, P. pfefferi, P. sergentorum, P. stellatum* [54] are incomplete. Information about morphology of gametocytes is absent. Molecular characterization is unavailable. Species identification is questionable based on the available information

^f^Paperna et al. [54] published re-description of *P. rouxi* from non-type avian host (*Alauda arvensis*, Alaudidae instead of *Passer hispaniolensis*, Passeridae whose is the type host). The re-description is based on samples, which were collected beyond of the type locality (France, instead of Algeria which is the type locality). This contradicts the Article 75.3.6 of the International Code of Zoological Nomenclature [78]. Additionally, according to [54], the erythrocytic meronts of the parasite from *A. arvensis* do not possess refractive globules and gametocytes possess few tiny pigment granules (Figs. 8, 9 in [54]). These are not characters of *P. rouxi*, which was described by Sergent et al. [111]. Sergent’s original material from Algiers labelled “2198, 26.4.28, Institut Pasteur d’Algérie” is available in the Natural History Museum, London. Examination of this blood film showed that numerous erythrocytic meronts of this parasite possess refractive globules (Fig. 3f, j) and gametocytes possess few large (Fig. 4o) pigment granules. The latter character is an important feature of *P. rouxi.* Based on available information, the parasite described in [54] as *‘P. rouxi’* cannot be attributed to *P. rouxi* and is considered as a *species inquirenda*. The parasite described by Paperna et al. [54] is characterized by presence of (1) the relatively prominent cytoplasm in growing meronts and (2) tiny size of pigment granules in gametocytes, so might belong to a new *Plasmodium* species. Additional investigation is needed to answer this question. In the same study, Paperna et al. [54] described a new species *Plasmodium mohammedi*, which was reported, *Passer domesticus* (the common host of *P. rouxi* in Mediterranian region [68]). Blood stages of *P. mohammed*i are indistinguishable from *P. rouxi* [111], particularly due to the presence of refractive globules in erythrocytic meronts and large pigment granules in gametocytes (see Figs. 18–21 in [54]). *Plasmodium mohammedi* is a synonym of *P. rouxi*. Molecular identification of *P. rouxi* (lineage pPADOM16) was developed [68]. Application of the barcoding indicates that the details of disposition of nuclei in erythrocytic meronts during different infections, particularly in different avian hosts, is variable in *P. rouxi*, but binuclear “bow-tie” form parasites often are present (Fig. 3f) and can be used for this parasite species identification. Additionally, presence of few large pigment granules in mature gametocytes also is a characteristic feature, and it recommended to use for distinguishing *P. rouxi* infection (Fig. 4o) from other Novyella parasites producing tetranuclear erythrocytic meronts

Additionally, in many of such parasite descriptions, gametocytes were not described, but this stage is essential for the identification of some *Plasmodium* species (Tables 3, 4, 5, 6, Figs. 4, 5). It is important to note that the descriptions of many *Plasmodium* parasites, which were incorporated in Table 9 and published during past 15 years, contain some information about their blood stages. Additionally, the type material was designated in many descriptions, but usually is insufficient for practical use and distinguishing parasites at the species level, particularly because (1) the type preparations contain co-infections and (2) single cells (meronts) were designated as holotypes. Single cells usually do not reflect entire morphological diversity of malaria parasites, so deposition of parahapantotype material is preferable in wildlife haemosporidian research [35, 49, 58, 78]. Validation of some names listed in Table 9 is possible in the future, but it requires additional research, preferably based on new samples from the same avian hosts and type localities.

Invalid *Plasmodium* parasite names (*nomen nudum*) are listed in Table 9. These names were not accompanied with descriptions so have no status in nomenclature. The names of this category can be used as a reserve for new parasite descriptions in the future, but it is preferable not to use them to avoid taxonomic confusion [78].

**Fig. 6:**
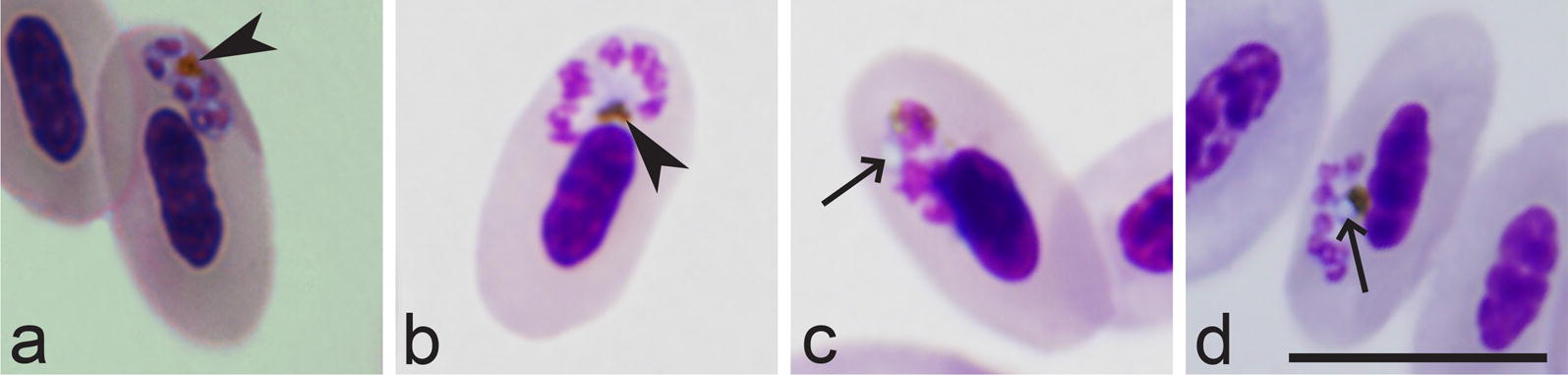
Maturing erythrocytic meronts of *Plasmodium ashfordi* (lineage pGRW2) in naturally infected the Common cuckoo *Cuculus canorus* (**a**) and experimentally infected Eurasia siskin *Carduelis spinus* (**b**–**d**) during the first (**b**) and 3–4th (**c**, **d**) passages of infected blood. Note that refractive globules were absent in erythrocytic meronts during the natural infection (**a**) and the first passage of the experimental infection (**b**), but develop in subsequent passages of the same strain in Eurasian siskin. Symbols are as in Figs. 1 and 3

The subgenus *Papernaia* was created for *Novyella*-like avian malaria parasites, whose erythrocytic meronts do not possess globules (Fig. 3f, h–l), structures of unclear origin and function [79, 80]. The feature of the presence or absence of such globules is used in distinguishing some species of malaria parasites belonging to subgenus *Novyella* during natural infections (Table 5). It is interesting to note that experimental studies with a *Plasmodium ashfordi* (pGRW2) strain, which normally do not possess globules in erythrocytic meronts, show that the globules appeared in this parasite’s meronts after several artificial passages in unusual avian hosts. This strain was originally isolated from the Common cuckoo *Cuculus canorus* (Cuculiformes), and it did not possessed globules in erythrocytic meronts [75] in the cuckoo or during the first passage in the Eurasian siskin *Carduelis spinus*. However, the globules appeared in the meronts of the same lineage after 3–4 passages via the infected blood inoculation in passeriform birds (G. Valkiūnas, unpublished). Molecular testing showed that the parasite lineage was the same. Pictures of erythrocytic meronts of the same isolate of the lineage pGRW2 in the Common cuckoo (Fig. 6a) and after the first passage in the Eurasian siskin *Carduelis spinus* (Passeriformes) (Fig. 6b) and several subsequent passages in siskins (Fig. 6c, d) illustrate this change. These experimental data indicate that malaria parasites which do not possess globules in natural hosts might develop this structure after artificial passages via infected blood inoculation in unusual avian hosts. In other words, this feature hardly can be used in taxonomy of avian *Plasmodium* parasites at subgenus level. It is preferable to limit use of the feature of absence or presence of globules in erythrocytic meronts to identification of natural infections at species level, on which the taxonomic validity of this feature also needs to be tested. Experimental sporozoite-induced infections of same parasites lineages possessing and not possessing globules in different avian hosts might help to answer the question about taxonomic value of this feature. Until additional information is available, *Papernaia* is considered as a synonym of subgenus *Novyella*.

## Conclusion

Based on available morphological data and DNA sequence information, 55 species of avian *Plasmodium* parasites can be readily distinguished. Species of subgenus *Novyella* predominate among them. Dichotomous keys for identification of these parasites were compiled allowing identification of these pathogens using morphological features of their blood stages. The majority of described avian *Plasmodium* species are mainly transmitted in countries with warm climates. The obstacles for their global spread remain insufficiently understood, mainly because of limited information on life cycles and vectors of the majority of described parasites of tropical birds. The lists of synonymous names as well as names of the categories *species inquirenda* and *incertae sedis* should be considered in future taxonomic work of avian malaria parasite at species level. The majority of described *Plasmodium* parasites have not been characterized using molecular markers, which development is an essential task for current avian malaria researchers.
